# Decrease in Blood Neutrophil-to-Lymphocyte Ratio Indicates Better Survival After Neoadjuvant Chemotherapy in Patients With Advanced Gastric Cancer

**DOI:** 10.3389/fsurg.2021.745748

**Published:** 2021-11-16

**Authors:** Ziyi Liu, Yahang Liang, Xiaolong Tang, Hui Qu

**Affiliations:** ^1^Department of Clinical Medicine, Qilu Medical College of Shandong University, Jinan, China; ^2^Department of General Surgery, Qilu Hospital of Shandong University, Jinan, China

**Keywords:** advanced gastric cancer, neutrophil-to-lymphocyte ratio, neoadjuvant chemotherapy, dynamic change, overall survival, disease-free survival

## Abstract

**Introduction:** Gastric cancer is the fifth most commonly diagnosed tumor and is the fourth leading cause of cancer-related mortality, worldwide. Due to the low rate of early diagnosis, approximately two-thirds of patients are first diagnosed at an advanced stage. Neoadjuvant chemotherapy (NAC) is recommended for patients with advanced gastric cancer (AGC). The neutrophil-to-lymphocyte ratio (NLR), a combined inflammatory and immunogenic factor, has been universally used for predicting outcomes in AGC patients. Given that NLR is a dynamic process, in this study, we investigated the value of NLR change for the prediction of chemotherapeutic responses and prognosis in patients with AGC.

**Methods:** We retrospectively enrolled 111 patients with AGC who underwent NAC following curative surgery. Patients were divided into two groups according to the NLR change after chemotherapy into the increased and decreased groups. Outcome measures were overall survival (OS) and disease-free survival (DFS). Univariate was calculated by Kaplan-Meier method. Multivariate analysis was performed using the Cox proportional hazards regression model.

**Results:** Post-chemotherapy, NLR increased in 36 patients and decreased in 75 patients. After a median follow-up time of 19 months, six patients developed local recurrence, 23 developed distant recurrence, and 34 died. Patients with reduced post-chemotherapy NLR showed significantly longer OS (*p* < 0.001) and DFS (*p* < 0.001). A decrease in the NLR after NAC was an independent indicator associated with better OS (*p* < 0.001) and DFS (*p* < 0.001).

**Conclusions:** In patients with AGC, a decrease in NLR after NAC indicated better survival. NLR change could serve as a robust indicator for the efficiency of NAC and prognostic prediction in patients with AGC.

## Introduction

Globally, over a million new cases and 769,000 deaths due to gastric cancer (GC) were diagnosed in 2020, making it the fifth most commonly diagnosed cancer and the fourth leading cause of cancer-related deaths. In particular, the incidence rate of GC is the highest in East Asia (1). Due to the absence of specific early symptoms in gastric cancer (2), ~70% of patients are first diagnosed in an advanced stage (3). Complete surgical resection remains the only curative method for patients with advanced gastric cancer (AGC) (4). However, even after adequate gastric resection, the 5-year survival rate remains at ~25% (5). To improve the prognosis of patients with AGC, multidisciplinary therapies including neoadjuvant chemotherapy (NAC), targeted therapy (6), surgery, immunotherapy, and chemoradiotherapy are recommended by the latest National Comprehensive Cancer Network (NCCN) guidelines (7).

To evaluate the effect of NAC, clinical response and post-operative histopathological examination have been widely used. But these evaluations are not comprehensive which can further influence the selection of post-operative therapeutic strategies. Therefore, other factors, such as inflammatory responses should be considered while evaluating the efficacy of NAC and identifying the patients who could benefit from NAC with potentially better prognosis.

The relationship between systemic inflammation and cancer progression has been generally studied (8–10). Several inflammatory markers, for instance, C-reactive protein (CRP), leukocytes, neutrophils, lymphocytes, monocytes, macrophages, and platelet counts are associated with various cancer types (11–15). Beyond these, the neutrophil-to-lymphocyte ratio (NLR) has gained increased attention in recent years. A meta-analysis comprising of 100 studies consisting of 40,559 patients shows that high NLR is associated with an adverse overall survival (OS) (HR = 1.81, 95% CI = 1.67 to 1.97; *p* < 0.001) in many solid tumors (16). NLR has also been universally identified as a promising prognostic factor for patients with AGC. Low pre-treatment NLR indicates a favorable prognosis for both OS and disease-free survival (DFS) (17, 18).

Most researchers emphasize the predictive value of NLR in patients with AGC who undergo curative gastrectomy, while only a few commit to the importance of NLR in patients receiving NAC. Additionally, current clinical studies have not reached a consensus on the specific value of NLR. The selection of parameters, such as median, mean, or the cutoff value varies in each article, which further weakens the application of NLR. However, to date, NLR remains dynamic (19). The change in NLR after chemotherapy can better reflect the process of tumor-related immune responses in the body. The purpose of this study was to investigate the applicability of NLR change in the prediction of chemotherapeutic response and prognosis in patients with AGC.

## Patients and Methods

### Patients

A total of 111 patients with AGC who underwent NAC and curative gastrectomy at the Department of Gastrointestinal Surgery, Affiliated Qilu Hospital, Shandong University, between August 2016 and December 2019 were enrolled retrospectively in this study. Pathological staging was performed according to the 8th edition of the American Joint Committee on Cancer (AJCC) Cancer Staging Manual. Patients with the following conditions were excluded: distant metastases or peritoneal implantation, previous chemotherapy or radiotherapy treatment, simultaneous malignancies other than GC, and recurrent or metastasis GC confirmed by histopathology or cytology. Post-operative telephonic follow-up was performed for 6 months to 3 years or till death.

### Treatment Regime

Before curative gastrectomy, patients were mainly administered with one of three chemotherapeutic regimens as follows: S-1 + oxaliplatin (SOX), oxaliplatin + capecitabine (XELOX), 5-Fu + leucovorin + oxaliplatin + docetaxel (mFLOT); SOX regimen: Oral tegafur–gimeracil oteracil potassium capsule (S-1) 100 mg/m^2^ on days 1–14, oxaliplatin 130 mg/m^2^ on day 1, followed by seven days of rest, and SOX regimen was repeated every 3 weeks for up to 3–8 cycles; XELOX regimen: oxaliplatin, 150 mg/m^2^, by intravenous infusion, on day 1, capecitabine, 1,000 mg/m^2^, orally, twice/day on days 1–14. XELOX was repeated every 3 weeks for up to 3–6 cycles. Lastly, mFLOT regimen: 24-h 5-FU 2200 mg/m^2^, leucovorin 200 mg/m^2^, oxaliplatin 75 mg/m^2^, docetaxel 45 mg/m^2^, by intravenous infusion, on day 1 of each 14-day cycle for up to 4 cycles.

### NLR Assessment

For all patients, a routine blood examination was performed at two stages: (1) Pre-chemotherapy: within 1 week before the initial day of chemotherapy; (2) post-chemotherapy: after finishing the last dose of chemotherapy and within 1 week before surgery. Patients were divided into two, namely, increased and decreased groups, based on their variations in NLR after NAC.

### Statistical Analysis

Clinical response was defined based on OS and DFS. OS was calculated from the first day of NAC till death due to any cause or last contact. DFS was calculated from the initial date of NAC until disease recurrence, death, or last contact. Clinicopathological characteristics were analyzed using the Chi-square test. OS and DFS were calculated by the Kaplan-Meier method and analyzed for significance by the log-rank test. Variables that were clinically relevant or showed a univariable association (*p* < 0.1) with the outcomes (DFS and OS) were included in the subsequent multivariate Cox proportional-hazard regression model. Given the number of available events, we carefully chose the variables for inclusion to ensure simplicity in the final model. All data were analyzed using the SPSS 25.0 statistical software. *P* < 0.05 was considered statistically significant.

## Results

### Clinicopathological Characteristics

#### Patient Characteristics

Table 1 shows the clinicopathological characteristics of the 111 enrolled patients (83 male; 28 female) with a median age of 58 years (range: 32–81 years).

**Table 1:** Clinicopathological features.

**Variables**	**Change of NLR**			***p*** **value**
	**Total (***n***)**	**Increasen** **(***n*** = 36)**	**Decrease** **(***n*** = 75)**	
Sex				0.150
Male	83	30	53	
Female	28	6	22	
Age (yr)				0.939
<60	58	19	39	
≥60	53	17	36	
Chemotherapy regimen				0.281
SOX	63	18	45	
XELOX	10	6	4	
FLOT	13	4	9	
Others	25	8	17	
Differentiation				0.130
High & medium	42	10	32	
Low, •signet-ring cell carcinoma, •mucinous adenocarcinoma	69	26	43	
Tumor size (cm)				0.939
<5	53	17	36	
≥5	58	19	39	
Tumor location				0.197
Upper 1/3	40	9	31	
Meddle 1/3	38	13	25	
Lower 1/3	33	14	19	
pT stage				0.739
1	7	2	5	
2	14	3	11	
3	48	18	30	
4	42	13	29	
Lymphatic metastasis				0.353
Yes	80	28	52	
No	31	8	23	
Venous invasion				0.920
Yes	47	15	32	
No	64	21	43	
Post-chemotherapy NLR				<0.001
<1.75	55	7	48	
≥1.75	56	29	27	
Recurrence				0.662
Yes	6	1	5	
No	105	35	70	
Metastasis				0.077
Yes	23	11	12	
No	88	25	63	
Outcome				<0.001
Alive	77	13	64	
Death	34	23	11	

*NLR, neutrophil-to-lymphocyte ratio; pT, pathological T stage; DFS, disease-free survival; CI, confidence interval; HR, hazard ratio*.

#### Blood Examination Data

The median pre-chemotherapy and post-chemotherapy NLR were 2.28 and 1.75, respectively. According to NLR change after chemotherapy, 36 patients were classified in the increased NLR group, while 75 patients were in the decreased NLR group.

#### Prognostic Values

A total of six patients showed local recurrence and 23 patients showed distant metastasis. Thirty four patients died during the follow-up. Among them, 28 patients had tumor-related deaths and six patients died due to multiple organ dysfunction syndrome (MODS) or other reasons.

## Comparison of Survival Outcome Between NLR Increased and Decreased Groups

Kaplan-Meier survival curve was plotted, and the log-rank test was used to analyze OS and DFS. NLR increased in 36 patients after chemotherapy, including 29 patients having an NLR greater than the median post-chemotherapy value of 1.75, while eight patients had NLR <1.75. NLR decreased in 75 patients after chemotherapy, with seven patients having an NLR >1.75, while 48 patients had an NLR <1.75.

According to the median post-chemotherapy NLR of 1.75 and the change in NLR after chemotherapy, all 111 patients were further divided into the following 4 subgroups: NLR ≥ 1.75 + decrease; NLR ≥ 1.75 + increase; NLR <1.75 + decrease; and NLR <1.75 + increase. By the end of the follow-up, the OS and DFS rates of NLR ≥ 1.75 + decrease group were 72.5 and 67.4%, respectively. In the NLR <1.75 + decrease group, the OS and DFS rates were 76.4 and 74.3%, respectively. The median OS and DFS were 14.0 months and 13.0 months, respectively, in the NLR ≥ 1.75 + increase group. In the NLR <1.75 + increase group, the median OS and DFS were 15.3 months and 14.3 months, respectively.

**Figure 1:**
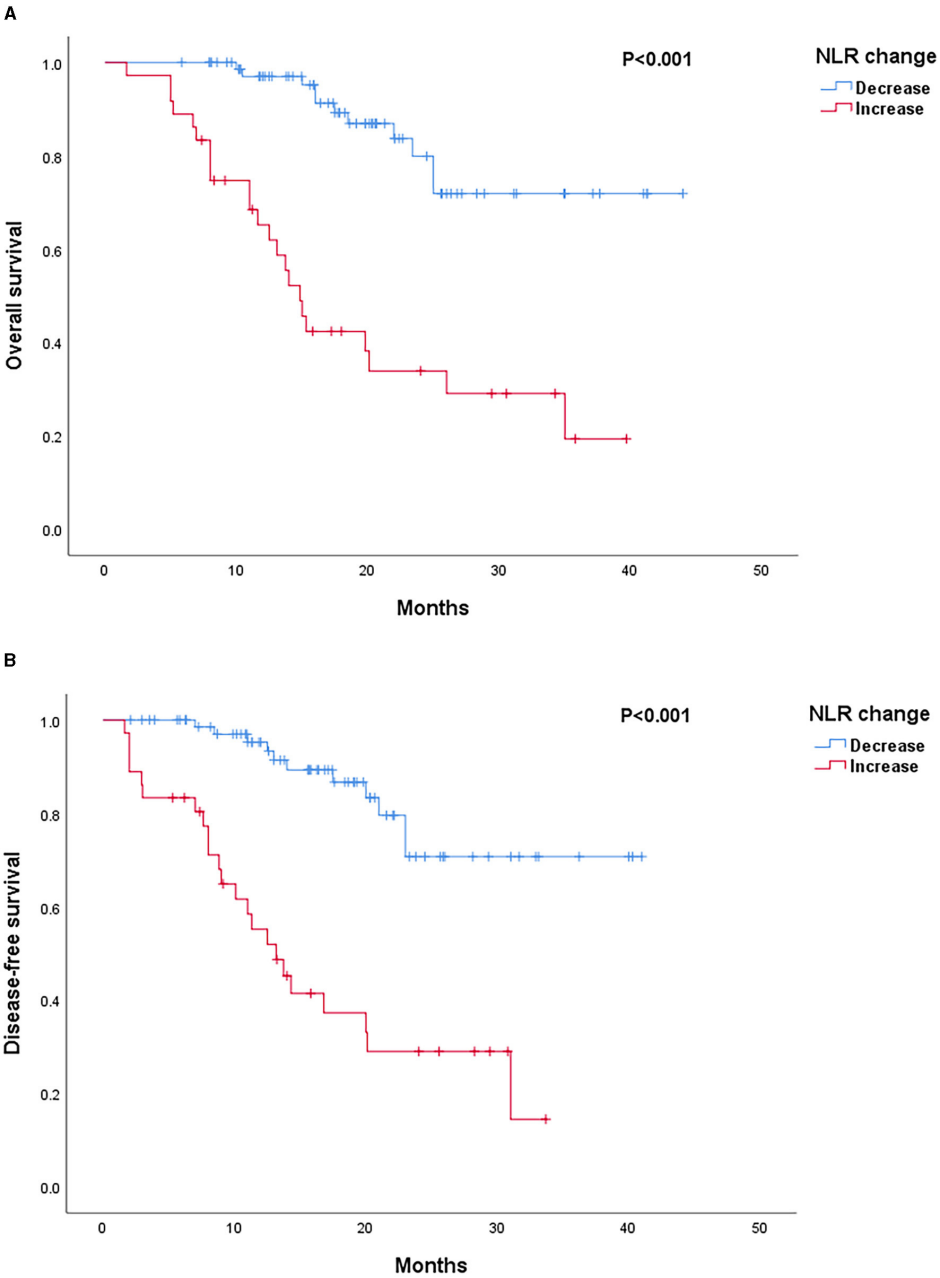
**(A)** Survival outcomes according to neutrophil-to-lymphocyte ratio (NLR) change. Kaplan–Meier curves of OS. **(B)** Survival outcomes according to NLR change. Kaplan–Meier curves of DFS.

**Figure 2:**
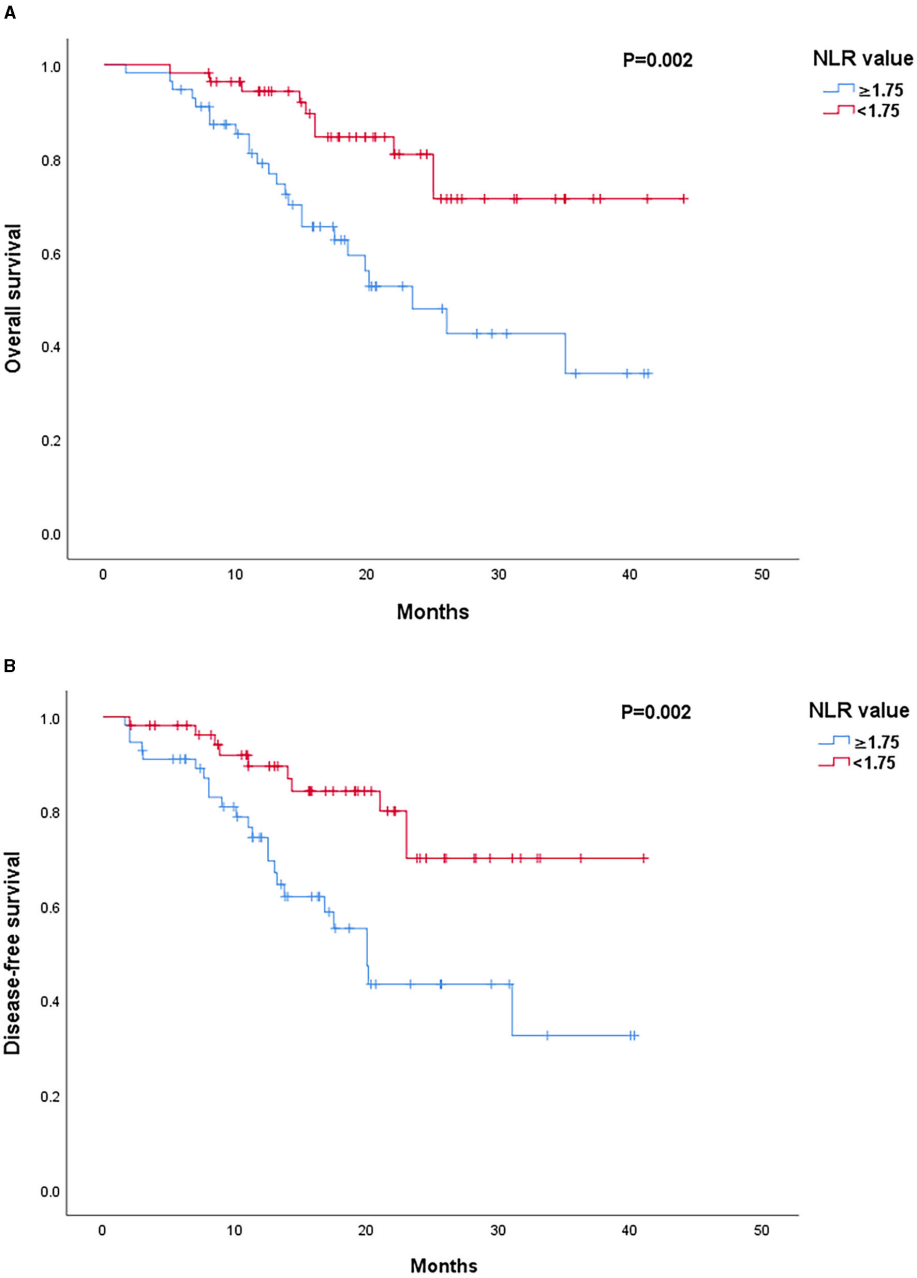
**(A)** Survival outcomes according to post-chemotherapy NLR value. Kaplan–Meier curves of OS. **(B)** Survival outcomes according to post-chemotherapy NLR value. Kaplan–Meier curves of DFS.

**Figure 3:**
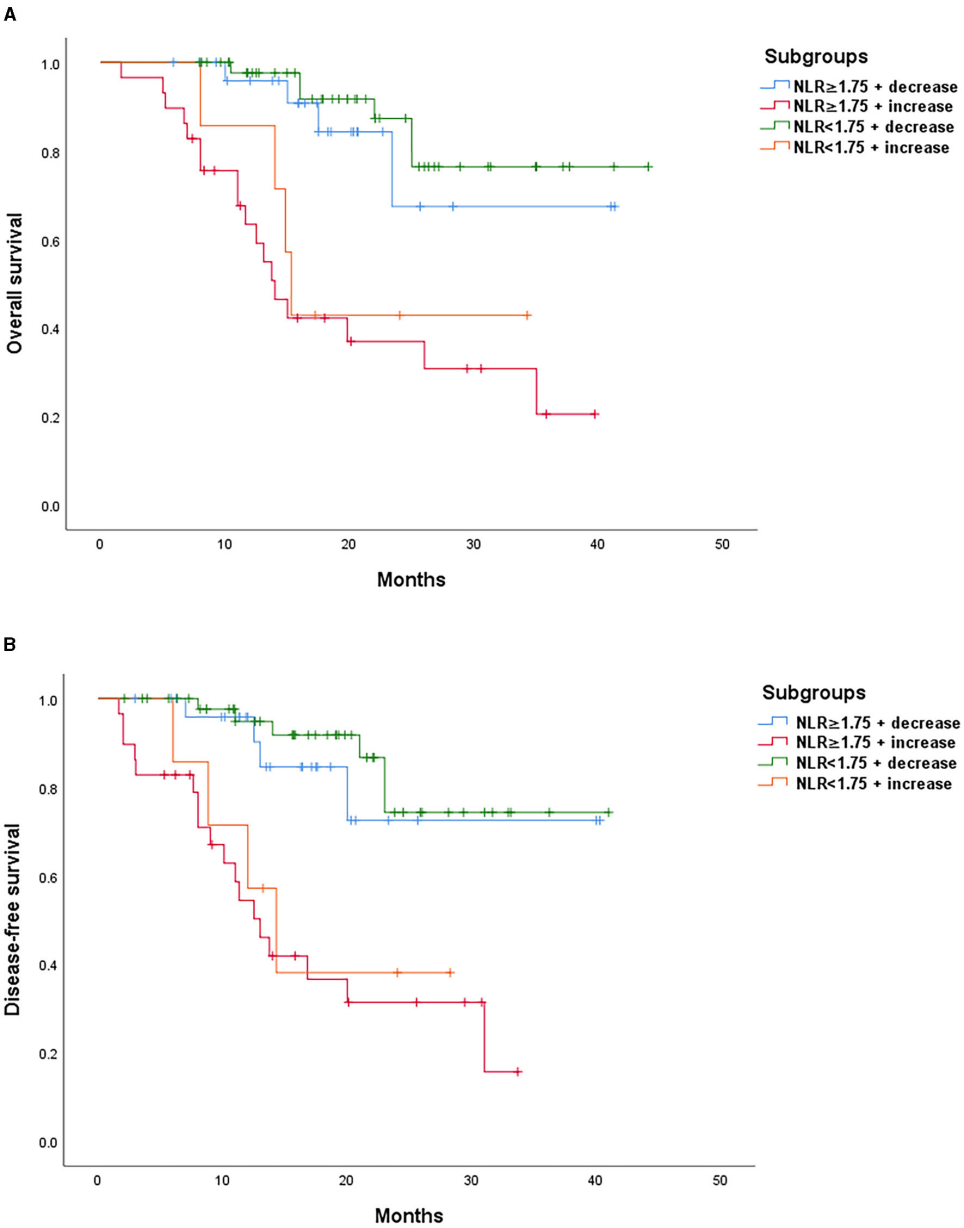
**(A)** Survival outcomes according to subgroups. Kaplan–Meier curves of OS. **(B)** Survival outcomes according to subgroups. Kaplan–Meier curves of DFS.

Patients with decreased NLR after NAC showed significantly favorable outcomes as compared to those with an increased NLR in both OS (*p* < 0.001) and DFS (*p* < 0.001) rates (Figure 1). Low post-chemotherapy NLR (<1.75) patients showed significantly longer OS (*p* = 0.002) and DFS (*p* = 0.002) than those with high post-chemotherapy NLR (≥1.75) (Figure 2). Interestingly, patients with high (≥1.75) but reduced post-chemotherapy NLR showed better OS (*p* = 0.036) and DFS (*p* = 0.039) than those with low (<1.75) but increased post-chemotherapy NLR (Figure 3). Among the high NLR (≥1.75) patients, those with decreased NLR showed longer OS (*p* = 0.001) and DFS (*p* = 0.001) than those with an increased NLR. Similarly, among the low NLR (<1.75) patients, those with decreased NLR showed longer OS (*p* = 0.001) and DFS (*p* = 0.005) than those with an increased NLR. As for the increased NLR group, there were no statistically significant differences between patients with high NLR (≥1.75) or low NLR (<1.75) in both OS (*p* = 0.519) and DFS (*p* = 0.672). Similar trends were observed in the decreased NLR group, and OS (*p* = 0.425) and DFS (*p* = 0.460) showed no significant differences among patients with high NLR (≥1.75) or low NLR (<1.75).

## Prognostic Factors

Prognostic factors for DFS and OS were analyzed using univariate and multivariate analyses based on the Kaplan-Meier method and Cox regression model, respectively. Univariate analysis for OS showed that differentiation (*p* = 0.029), tumor location (*p* = 0.004), venous invasion (*p* = 0.039), metastasis (*p* < 0.001), post-chemotherapy NLR value (*p* = 0.002), and NLR change (*p* < 0.001) were significant risk factors (Table 2). Additionally, the significant risk factors for DFS were differentiation (*p* = 0.024), tumor location (*p* = 0.003), venous invasion (*p* = 0.034), metastasis (*p* < 0.001), post-chemotherapy NLR value (*p* = 0.002), and NLR change (*p* < 0.001, Table 3).

**Table 2:** Univariate and multivariate analysis for overall survival (OS) in patients with advanced gastric cancer (AGC), who received neoadjuvant chemotherapy (NAC).

	**Univariate**	**Multivariate**		
**Variables**	***p*** **-value**	**HR**	**95%CI**	***p*** **-value**
Sex	0.408			
Male/female				
Age (yr)	0.413			
<60/≥60				
Chemotherapy regimen	0.486			
SOX/XELOX/FLOT/others				
Differentiation	0.029	0.485	0.246–1.537	0.299
High & medium/Low,• signet-ring cell carcinoma,• mucinous adenocarcinoma				
Tumor size (cm)	0.714			
<5/≥5				
Tumor location	0.004	1.127	0.981–9.713	0.054
Upper/meddle/lower				
pT stage	0.454			
1/2/3/4				
Lymphatic metastasis	0.698			
Yes/no				
Venous invasion	0.039	0.473	0.289–1.342	0.227
Yes/no				
Recurrence	0.168			
Yes/no				
Metastasis	<0.001	1.920	0.062–0.345	<0.001
Yes/no				
Pre-chemotherapy NLR	0.403			
<2.28/≥2.28				
Post-chemotherapy NLR	0.002	0.516	0.202–1.763	0.350
<1.75/≥1.75				
Change of NLR	<0.001	1.945	2.180–22.438	0.001
Increase/decrease				

*NLR, neutrophil-to-lymphocyte ratio; pT, pathological T stage; DFS, disease-free survival; CI, confidence interval; HR, hazard ratio*.

**Table 3:** Univariate and multivariate analysis for disease-free survival (DFS) in patients with AGC, who received NAC.

	**Univariate**	**Multivariate**		
**Variables**	***p*** **-value**	**HR**	**95%CI**	***p*** **-value**
Sex	0.496			
Male/female				
Age (yr)	0.482			
<60/≥60				
Chemotherapy regimen	0.469			
SOX/XELOX/FLOT/others				
Differentiation	0.024	0.661	0.208–1.280	0.153
High & medium/low,• signet-ring cell carcinoma,• mucinous adenocarcinoma				
Tumor size (cm)	0.797			
<5/≥5				
Tumor location	0.003	0.720	0.679–6.218	0.203
Upper/meddle/lower				
pT stage	0.454			
1/2/3/4				
Lymphatic metastasis	0.660			
Yes/no				
Venous invasion	0.034	0.440	0.294–1.410	0.271
Yes/no				
Recurrence	0.131			
Yes/no				
Metastasis	<0.001	1.742	0.075–0.407	<0.001
Yes/no				
Pre-chemotherapy NLR	0.357			
<2.28/≥2.28				
Post-chemotherapy NLR	0.002	0.511	0.199–1.813	0.365
<1.75/≥1.75				
Change of NLR	<0.001	1.753	1.861–17.900	0.002
Increase/decrease				

*NLR, neutrophil-to-lymphocyte ratio; pT, pathological T stage; DFS, disease-free survival; CI, confidence interval; HR, hazard ratio*.

Factors that showed a univariable association (*p* < 0.1) with DFS or OS were included further in the multivariate Cox proportional-hazard regression model. According to multivariate analysis, metastasis (HR 0.147, 95% CI 0.062–0.345; *p* < 0.001) and NLR change (HR 1.920, 95% CI 2.180–22.438; *p* < 0.001) were independently associated with poor OS (Table 2), metastasis (HR 1.742, 95% CI 0.075–0.407; *p* < 0.001), and NLR change (HR 1.753, 95% CI 1.861–17.900; *p* < 0.001) were also independently associated with poor DFS (Table 3).

## Discussion

In this study, we investigated the prognostic value of NLR change in 111 patients with AGC who had received NAC. Patients were grouped according to the change in NLR after chemotherapy, and further sub-grouped according to their post-chemotherapy values of NLR. The results showed that regardless of whether their NLR was high (≥1.75) or low (<1.75) after chemotherapy, the survival in patients with decreased NLR after NAC was significantly better as compared to those with increased NLR (*p* < 0.001). Interestingly, patients with decreased and high post-chemotherapy NLR had significantly longer OS (*p* = 0.036) and DFS (*p* = 0.039) than those with increased and low post-chemotherapy NLR, even if their NLR value were greater than the median value of 1.75. Decreased NLR after NAC could function as an independent prognostic indicator of better OS (*p* < 0.001) and DFS (*p* < 0.001). We confirmed that a decrease in NLR after NAC indicated better survival in patients with AGC. More importantly, NLR change could serve as a robust biomarker for the evaluation of chemotherapeutic response and outcomes in patients with AGC, which could further help in the selection of subsequent treatment strategies. To our best knowledge, this is the first study that proved the prognostic significance of NLR change after NAC in patients with AGC.

Since the relationship between inflammation and cancer was put forward by Rudolf Virchow over 150 years ago (20), the role of inflammation in the pathogenesis and development of malignant neoplasms has been widely studied for several tumors (21–23). There is a complex interaction between tumor-host immunity and inflammatory response. The damage to tissue due to tumors can trigger a series of immune responses both locally and in the whole body. Although the accumulation of various immune cell types in tumors represent part of the anti-tumor response, they, in turn, are repolarized for pro-tumorigenic functions, including the inhibition of cytotoxic immune-cell recruitment (24).

NLR, a combination biomarker, reflects the inflammatory response. In cancer, neutrophils are a vital component of the tumor microenvironment (25) that facilitate tumor progression by provoking mutation in tumor suppressor genes, driving angiogenesis, secreting enzymes and cytokines to promote proliferation and metastasis, remodeling extracellular matrix, and immunosuppression (26, 27). While, lymphocytes, triggered by the systemic inflammatory response, expose depression in innate cellular immunity as indicated by the increase in T8 suppressor lymphocytes and a decrease in T4 helper lymphocytes (28). Consequently, high NLR promotes tumor growth and inhibits anti-tumor response, ultimately leading to unfavorable prognosis in patients.

Although the role of NLR in predicting survival in patients with AGC has been confirmed in previous studies (29–31), the clinical values of NLR remain unclear in patients with AGC who have undergone NAC. Another non-negligible limitation in the existing studies is that the selection of NLR value parameters, such as median, mean, or the cutoff value derived from the ROC curve, varies between articles, which further weakens the applicability of NLR due to the lack of a uniform standard. NLR is a dynamic process. When inflammatory reactions and immune responses of the body are imbalanced, the anti-tumor action weakens and tumor growth-promoting effects are seen. The change in NLR is reflective of the transformation of this balance. Therefore, we bridged the NLR change with tumor outcomes and NAC efficiency in this study. Two key findings have emerged as follows: first, the decrease in NLR after NAC indicated resolution of inflammation or better chemotherapeutic response; second, decreased NLR after NAC implied better survival in patients with AGC. Our research addressed some previous limitations of existing studies and provided convenient and meaningful information for clinical practice. Theoretically, NLR change as an inflammation marker can better reflect the dynamic processes in the body. Clinically, NLR change can help in evaluating the efficiency of NAC. Prognostic stratification based on NLR change may guide physicians to incorporate a specific treatment regimen based on the performance of the patient. For example, drugs may be re-prescribed for targeted action in the poor prognosis group. Further studies are needed to validate this speculation. Additionally, early diagnosis and multidisciplinary therapies would be research hotspots in the future. NLR combined with traditional tumor markers and examinations may benefit to early screening outcomes in patients with GC. Besides, NLR change reflects the balance between inflammatory and immune responses in AGC patients. Thus, our study could provide strong evidence for the development of immunotherapy.

However, some limitations remain unaddressed in our study. First, this was a retrospective study at a single institution that enrolled a smaller number of patients. It is hard to draw a generalized conclusion as the populations in the present study do not represent all patients with AGC. Second, uniform NAC regimens and cycles were not administered to all patients enrolled in this study. Although more than 40% of the patients underwent a SOX treatment regimen, other regimens were also recommended. Failing to identify this information accurately could lead to a selection bias. Finally, we did not consider drug administration history which could influence the immune status (e.g., aspirin, NSAIDs, and corticosteroids). Hence, prospective clinical trials, multi-institutional enrolment, adequate sample sizes, and a single chemotherapeutic regimen should be considered in future investigations.

## Conclusion

In conclusion, NLR change reflects the dynamicity between tumor inflammatory and immune responses. Decrease in NLR value after NAC corresponded to a good chemotherapeutic response and improved survival rates. Patients with AGC with decreased NLR after NAC showed more favorable OS and DFS. We confirmed that NLR change was a reliable and easily available prognostic factor for patients with AGC who had undergone NAC.

## Data Availability Statement

The raw data supporting the conclusions of this article will be made available by the authors, without undue reservation.

## Ethics Statement

Written informed consent was obtained from the individual(s) for the publication of any potentially identifiable images or data included in this article.

## Author Contributions

ZL: investigation, resources, visualization, roles and writing—original draft, and writing—review and editing. YL: formal analysis and software. XT: data curation and project administration. HQ: conceptualization, funding acquisition, methodology, supervision, and validation. All authors contributed to the article and approved the submitted version.

## Conflict of Interest

The authors declare that the research was conducted in the absence of any commercial or financial relationships that could be construed as a potential conflict of interest.

## Publisher's Note

All claims expressed in this article are solely those of the authors and do not necessarily represent those of their affiliated organizations, or those of the publisher, the editors and the reviewers. Any product that may be evaluated in this article, or claim that may be made by its manufacturer, is not guaranteed or endorsed by the publisher.

## Abbreviations

AGC: advanced gastric cancer
NAC: neoadjuvant chemotherapy
NLR: neutrophil-to-lymphocyte ratio
DFS: disease-free survival
OS: overall survival
AJCC: American Joint Committee on Cancer
NCCN: National Comprehensive Cancer Network
MODS: multiple organ dysfunction syndrome.
